# 继发于泛发性淋巴管异常的慢性弥散性血管内凝血1例

**DOI:** 10.3760/cma.j.cn121090-20241202-00520

**Published:** 2025-10

**Authors:** 学敏 高, 路 张

**Affiliations:** 1 中国医学科学院北京协和医院内科，北京 100730 Departement of Internal Medicine, Peking Union Medical College Hospital, Chinese Academy of Medical Sciences and Peking Union Medical College, Beijing 100730, China; 2 中国医学科学院北京协和医院血液内科，北京 100730 Department of Hematology, Peking Union Medical College Hospital, Chinese Academy of Medical Sciences and Peking Union Medical College, Beijing 100730, China

患者，男，32岁，因“发现纤维蛋白原降低14年，便血10年”于2021年10月18日就诊于北京协和医院血液科。患者于2007年体检查血常规示PLT 195×10^9^/L，凝血功能提示凝血酶原时间（PT）16.2 s，活化部分凝血活酶时间（APTT）45 s，纤维蛋白原（FIB）0.73 g/L，D-二聚体（D-Dimer）、纤维蛋白降解产物（FDP）结果不详。胸部CT提示双肺门轮廓增大，支气管血管束增粗，小叶间隔增厚，胸椎旁软组织增厚，多个胸椎椎体及肋骨密度不均。进一步完善支气管镜提示双肺各叶、段支气管黏膜轻度增厚，以右中叶支气管为著，表面粗糙不平，轻度充血水肿。于右中叶支气管开口行黏膜活检，病理提示支气管平滑肌增生，黏膜下局部淋巴管扩张，符合淋巴管瘤病样改变，未进一步治疗，2007年至2011年患者无不适。2011年至2012年患者开始间断出现大便表面覆盖鲜血，2～3次每天。2012年7月患者曾咯鲜血10 ml，于当地医院化验示PLT 88×10^9^/L，凝血功能结果不详，胸部CT大致同2007年，经抗感染、低分子肝素6 400 U治疗后好转。2012年至2019年期间患者便次增加至6～7次每天，间断出现大便表面覆盖鲜血，量约10 ml/次，频率由每2～3个月1次增加至每个月2次，可自行好转；同期出现刷牙后牙龈出血、拔牙后出血不止，输注血浆后止血。患者2014年行肠镜提示乙状结肠、直肠弥漫分布出血斑。2019年12月患者再次就诊当地医院，复查PLT 112×10^9^/L，凝血功能：PT 13.7 s，APTT 34.3 s，FIB 0.76 g/L，D-Dimer 29.49 mg/L［纤维蛋白原等价单位（FEU）］，FDP 103.73 µg/ml，抗凝血酶Ⅲ 61.7％。肠镜提示乙状结肠、直肠黏膜下广泛血管扩张，大量片状红斑及点状糜烂。未进一步治疗。2019年至2020年患者曾无明显诱因出现腰部剧烈疼痛，活动后加重，后自行缓解，就诊于当地医院行X线检查提示L1椎体压缩性骨折，MRI提示多发椎体骨质密度不均；L1椎体穿刺活检病理提示少量骨及骨组织，部分造血细胞形态不规则。予伊班膦酸钠、补钙、补维生素D治疗，腰痛较前好转。2021年8月患者便血量增加至20～30 ml/次，就诊于当地医院查HGB 117 g/L，PLT 123×10^9^/L，FIB 0.52 g/L，D-Dimer 44.52 mg/L FEU，FIB抗原定量及活性均降低；CT提示肺部及纵隔病变大致同前，肝脏实质密度弥漫性轻度增高，不排除含铁血黄素沉积；PET-CT提示椎旁软组织为髓外造血可能；当地医院予患者输注FIB、冷沉淀凝血因子、血浆后症状好转。2021年9月13日患者就诊于我院，化验结果示PLT 121×10^9^/L，PT 13.9 s，APTT 31.9 s，FIB 0.82 g/L，D-Dimer 32.26 mg/L FEU，遗传性出凝血疾病DNA测序未见异常。考虑慢性弥散性血管内凝血（DIC）可能，收入我院血液科。入院查体：全身皮肤黏膜未见出血点及瘀斑，手指、足趾均为杵状指。脊柱胸椎段右侧弯，骶尾部压痛。

完善FIB纠正试验：予患者FIB 50 mg/kg输注，24 h后复查FIB水平，输注前后FIB分别为0.79 g/L、1.09 g/L，升高幅度≤1.0 g/L，且D-Dimer由27.97 mg/L FEU升高至44 mg/L FEU，FDP由79.6 µg/ml升高至118.3 µg/ml，提示FIB消耗增多，国际血栓与止血学会（ISTH）DIC评分4分，考虑非显性DIC，支持慢性DIC诊断。但DIC的诊断需要存在可能引起DIC的基础疾病，结合患者慢性病程及既往PET-CT等影像学结果，肿瘤和血管瘤可能性小，且患者无明确慢性感染证据。患者增强CT可见支气管血管束增粗，椎旁软组织密度影，伴多发中轴骨溶骨性病变，椎旁、肠系膜区、胸膜外与脊柱间脂肪间隙、骶前、双侧腹股沟区多发软组织密度影，腹膜后间隙多发迂曲管状影。全身MRI提示多发中轴骨、椎旁软组织、纵隔间隙内、双侧支气管血管束周围、双侧胸膜区、肝门区、肝脏Glisson鞘、腹膜后间隙、肠系膜区、骶前间隙、骶骨周围软组织、双侧腹股沟区弥漫多发T2WI异常高信号影，考虑脉管来源病变。患者外院右支气管开口处黏膜活检于我院病理会诊见支气管黏膜内较多不规则扩张的淋巴管。考虑患者泛发性淋巴管异常（GLA）诊断明确。患者外周血未发现PI3K、BRAF相关通路基因突变。遂予患者低分子肝素4 000 U，每12 h 1次，皮下注射抗凝治疗，复查FIB逐渐回升至1.35 g/L，出血症状好转，后将低分子肝素加量至6 000 U每12 h 1次。原发病方面，予西罗莫司1 mg每日1次治疗。出院前复查PLT 140×10^9^/L，FIB 1.26 g/L，D-Dimer 17.55 mg/L FEU，FDP 48.9 µg/ml。患者病情好转，于2021年11月出院。患者出院时将低分子肝素改为利伐沙班20 mg每日1次抗凝治疗，出院1周后复查FIB回升至1.7 g/L。出院后患者因出血症状好转，未规律服药，自行停用西罗莫司及利伐沙班且未随诊。2023年因肺泡出血、呼吸衰竭去世。

讨论：GLA在2018年国际脉管性疾病研究学会的脉管性疾病分类中属于慢血流血管畸形亚类，该类疾病引起凝血功能异常的机制可能与异常的内皮细胞和异常的缓慢血流导致凝血系统异常激活有关，从而引起局限性血管内凝血，当病变部位多时，消耗性凝血病的表现更加突出。对于慢性DIC患者，需要在针对DIC进行抗凝治疗的同时，深入探究其背后的基础病因。除肿瘤、外伤、妊娠等常见的引起慢性DIC的病因外，临床医师在鉴别诊断时需注意纳入淋巴管异常这一罕见病因，同时重视抗凝治疗对此类患者的重要价值。

